# Novel vaccines targeting dendritic cells by coupling allergoids to mannan

**DOI:** 10.1007/s40629-018-0069-8

**Published:** 2018-05-18

**Authors:** Cristina Benito-Villalvilla, Irene Soria, José Luis Subiza, Oscar Palomares

**Affiliations:** 1 Department of Biochemistry and Molecular Biology, School of Chemistry, Complutense University of Madrid, 28040 Madrid, Spain; 2 Inmunotek, S.L., Alcalá de Henares, Spain

**Keywords:** Allergen-specific immunotherapy, Immune tolerance, Allergen vaccines, Allergoids conjugated to mannan, Dendritic cells, Regulatory T cells

## Abstract

Allergen-specific immunotherapy (AIT) is the single disease-modifying treatment for allergy. Clinical trials show AIT to be safe and effective for many patients; however, it still faces problems related to efficacy, safety, long treatment duration and low patient adherence. There has been intensive research to develop alternative strategies, including novel administration routes, adjuvants or hypoallergenic molecules. Promising results are reported for some of them, but clinical progress is still moderate. Allergoids conjugated to nonoxidized mannan from *Saccharomyces cerevisiae* have emerged as a novel concept of vaccine targeting dendritic cells (DCs). Preclinical human and animal models demonstrated that allergoids conjugated to mannan enhance allergen uptake, promote healthy responses to allergens by inducing Th1 and T regulatory (Treg) cells, and show clinical efficacy in veterinary medicine. Dose-finding phase II clinical trials in humans are currently ongoing. We review the current stage of allergoids conjugated to mannan as next generation vaccines for AIT.

## Introduction

**Fig. 1:**
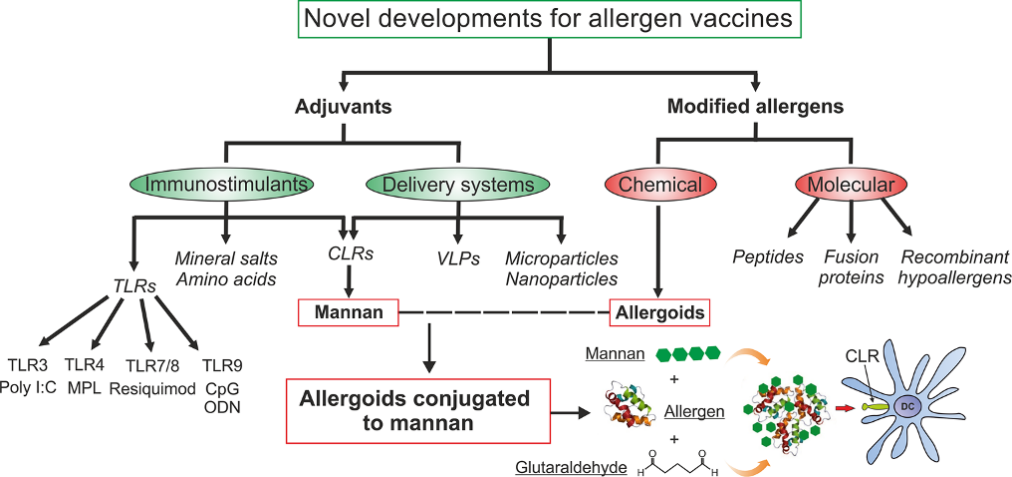
Novel developments for allergen vaccines. Adjuvants can be classified as immunostimulant substances including TLR-ligands, mineral salts, amino-acids or CLR-ligands and as delivery systems including micro- or nanoparticles, VLPs or CLR-ligands. There are different ways to modify allergens to make them hypoallergenic: molecular (peptides, fusion proteins or recombinant hypoallergens) or chemical (generating allergoids) strategies. Both approaches are used in combination to generate allergoids conjugated to mannan. *TLR* Toll-like receptor; *CLR* C-type lectin receptor; *VLP* Viral-like particle; *MPL* Monophosphoryl lipid A; *ODN* Oligodeoxynucleotide

Allergen-specific immunotherapy (AIT) is the only potential curative treatment for allergic diseases [1, 2]. AIT is based on the administration of high doses of the causative allergens to induce a state of permanent tolerance after treatment discontinuation. Currently, AIT is administered either subcutaneously or sublingually to restore healthy immune responses to allergens [3, 4]. Although many clinical trials and real-life experience show AIT as a safe and effective treatment for many allergic patients, it still faces important drawbacks. AIT remains underused mostly due to scattered availability of regimes and/or products for application, a lack of awareness, limited access to specialist care or the reimbursement policy [2, 5]. In addition, patient adherence is low, possibly due to the long duration and the large number of administrations needed to reach clinical efficacy [1]. Therefore, there is an urgent need to develop safer and more effective allergen vaccines that might help to overcome such drawbacks. In this regard, novel adjuvants or modified hypoallergenic variants have been investigated (Fig. 1; [6]). Adjuvants are substances capable of potentiating the therapeutic effect of an allergen vaccine without being an allergen per se [3, 6]. Proper AIT adjuvants must be safe, stable and promote Th1 and/or regulatory T (Treg) cell responses [3, 6–9]. In recent years, intensive research has been performed to develop novel adjuvants that can potentiate proper immune responses in target cells (immunopotentiators) or increase the capture/presentation of allergens by antigen presenting cells (delivery systems; [3, 6, 10]). Among immunopotentiators, different Toll-like receptor (TLR) agonists (poly I:C, monophosphoryl lipid A, resiquimod or unmethylated CpG motifs), mineral salts (aluminum hydroxide and others) or different variants of the amino acid tyrosine has been assayed with different success in AIT (Fig. 1). Similarly, several delivery systems such as viral like particles (VLP), micro- and nanoparticles have been developed. Among the strategies to obtain modified allergens, chemical modification of allergens to generate allergoids is by far the most successful one, being that these products are currently used for AIT in the clinical practice. Other strategies to generate hypoallergenic variants that are in different stages of development include peptides, fusion proteins or recombinant derivatives [7, 10–14].

Dendritic cells (DCs) play a key role in all the phases of allergy as well as in the induction of tolerance to allergens [15]. A plethora of studies demonstrated that DCs are essential in linking innate and adaptive immune responses and that different substances, cells, and surrounding signals can modulate the function of DCs [16–21]. Therefore, the design of novel vaccines targeting DCs has emerged as a useful approach to improve current AIT strategies [3, 18, 22]. In this regard, next generation vaccines formulated by coupling allergoids to nonoxidized mannan and targeting DCs through C‑type lectin receptors (CLR) represent a promising strategy for AIT ([18, 23]; Fig. 1). Herein, we will review and provide an update of the current stage of allergoids conjugated to mannan as novel vaccines targeting DCs for AIT. We will summarize and discuss the available data regarding the proof of concept, the production and characterization, the preclinical *in vitro* and *in vivo* human and experimental animal models as well as the current ongoing human clinical trials.

## Production and characterization of allergoids conjugated to nonoxidized mannan

Chemically modified allergens (allergoids) are hypoallergenic preparations widely used in AIT due to their excellent safety profile and clinical efficacy [10]. Moreover, their low reactivity with IgE makes these allergen preparations less prone to be captured by DCs through IgE-dependent mechanisms that facilitate Th2-biased responses [3, 24].

**Fig. 2:**
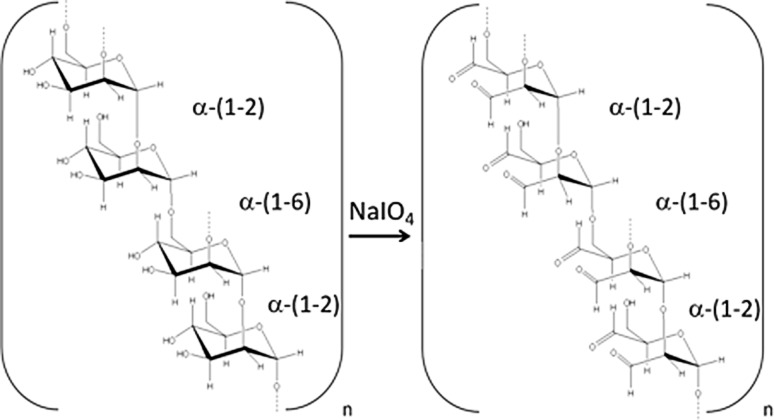
Sugar oxidation of mannan carbohydrate backbone. Mannan oxidation with sodium periodate (*NaIO*_*4*_) breaks the mannopyranose rings within the polymannose backbone between the adjacent hydroxyl groups at positions C3 and C4 generating highly reactive and unstable aldehyde groups

**Fig. 3:**
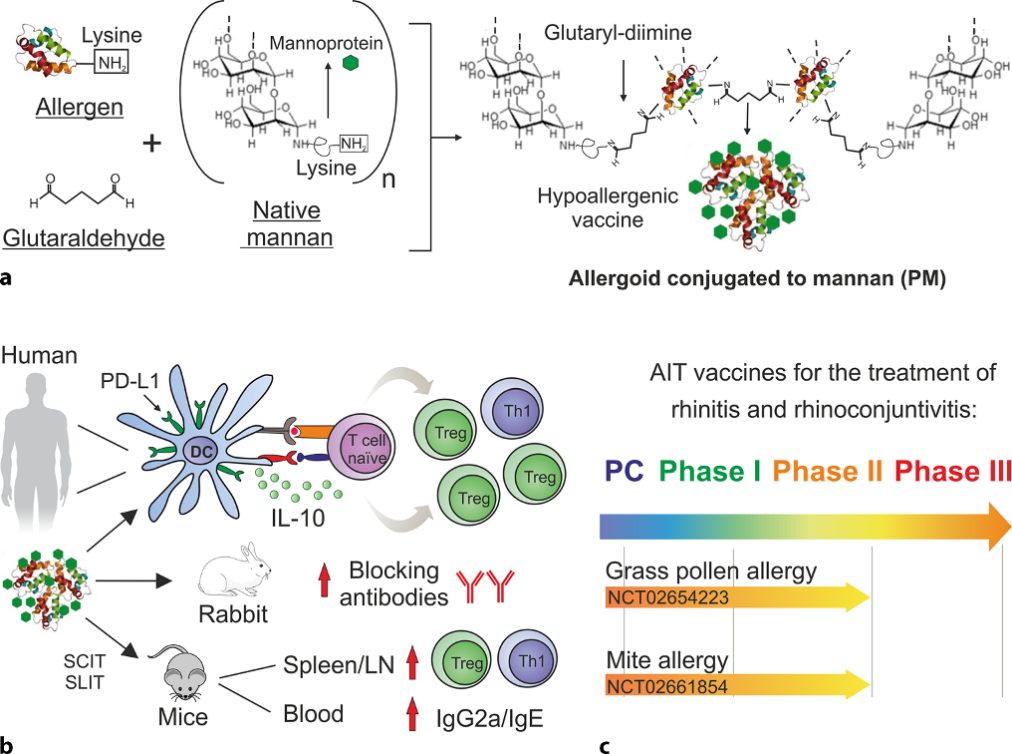
Steps for the generation and development of allergoids conjugated to mannan as vaccines for AIT. **a** Allergens are polymerized and conjugated with mannan under non-oxidative conditions in a single step using glutaraldehyde. **b** Preclinical experiments in human, rabbit, and mice models to establish the *in vivo* properties of allergoids conjugated to mannan (PM). **c** Dose-finding clinical trials for grass pollen and mite allergy using PM are currently ongoing. *LN* lymph nodes; *PC* Preclinical phase; *SCIT* subcutaneous immunotherapy; *SLIT* sublingual immunotherapy

Most allergoids are made by treating the native allergens with aldehydes (formaldehyde or glutaraldehyde) that react with the primary amine groups of lysines. Because of this fact, the accessibility of these groups is dramatically impaired in allergoids, making them unsuitable to be bound to mannan by conventional oxidative procedures. Actually, mannan oxidation generates aldehyde groups (Fig. 2) that react with the lysines contained in the allergen proteins but that are not available in allergoids for chemical conjugation. Therefore, to produce allergoids conjugated to mannan, an alternative approach was used taking advantage of three facts: (1) mannan can be purified from the yeast cell wall keeping the mannoprotein associated with the sugar backbone; (2) this mannoprotein contains a significant number of lysine residues; (3) glutaraldehyde is a di-aldehyde capable of reacting with two lysines from different molecules. In this way, glutaraldehyde could cross-link allergen and mannan molecules by means of a glutaryl-diimine group formed as the result of the reaction, which acts as a linker (Fig. 3a). On the other hand, glutaraldehyde in aqueous medium is present in a polymeric form in a certain proportion, allowing the crosslinking among several molecules, thus, favoring the creation of high molecular weight polymerized structures (Fig. 3a).

The suitability of coupling allergoids to mannan by means of glutaraldehyde has been demonstrated for pollen [18, 23, 25] and mite [26] allergens. By using this methodology a remarkable structural stability of allergoids conjugated to mannan is achieved [25], which is an important issue from the perspective of vaccine development. It should be noted that although oxidized mannan was not used with allergoids for the above technical reasons, the structural and chemical properties of nonoxidized (native) and oxidized mannan are completely different and, therefore, they should not be considered as interchangeable substances (Fig. 2; [18, 23]). As shown in Fig. 2, mild oxidation of mannan cleaves bonds between adjacent carbon atoms at position C3 and C4 containing hydroxyl groups (OH), thus, leading to the generation of highly reactive aldehydes. Such chemical cleavage dramatically affects the structural integrity of mannan as well as some of its key biological features, such as the binding capacity to certain mannan-binding lectins [27, 28]. Although it could be argued that the loss of mannan structural integrity may be minimized by reducing the degree of oxidation [29], the efficiency for conjugation under milder conditions might well be subjected to the nature of the specific composition of the allergen extracts at the protein level [30]. Remarkably, the oxidation/reduction state of mannan significantly alters its biological properties on DCs, thus, conditioning the functional behavior of these cells upon mannan stimulation [31]. It is also noteworthy to mention that when using glutaraldehyde for conjugation, the protein coupling takes place through the peptide tail present in the mannoprotein, thus, keeping free the poly-mannose backbone (Fig. 3a). This might well result in a better accessibility of mannoses to the cell surface receptors on DCs due to the reduction of the potential steric hindrance of the bound allergen.

The origin and nature of the mannan used for coupling is also an important issue to consider. Cell wall mannan from different yeasts differs in structure [28] and biological activity on DCs [32]. For example, by using nonoxidized mannan derived from *S. cerevisae*, no induction of Th17 responses by DCs was detected [18], in line with other reports [33] and in contrast to mannan derived from *C. albicans* [33]. Recent studies have demonstrated that Bet v 1 conjugated to oxidized mannan from *S. cerevisae* induced strong Th1 but also Th17 responses [34], thus, resembling immune responses triggered by mannan from *C. albicans *[33].

## Preclinical assessment of allergoids conjugated to mannan in human models

To assess the potential of these novel glycoconjugate-allergoids to be used as next generation vaccines for AIT, different preclinical human and animal models were employed. As a proof of concept, allergoids conjugated to nonoxidized mannan from *S. cerevisiae *using *Phleum pratense* grass pollen allergens were produced as shown in Fig. 3a [25]. These polymerized allergoid-mannan structures, that we will call PM from here onwards, are hypoallergenic as they display low *in vitro* IgE-binding reactivity, reduced capacity to activate *in vivo* mast cells by skin prick test as well as *ex vivo* basophils from grass pollen allergic patients [18]. Kinetic uptake studies performed in human monocyte-derived DCs (hmoDCs) using equally labelled native *P. pratense *(N), polymerized allergoids (P) or PM, revealed that PM are rapidly captured and in a much more efficient manner than N and P by hmoDCs [18]. The PM uptake by hmoDCs was partially inhibited, around 45%, in the presence of phenylarsine oxide, indicating that the process is mainly depending on receptor-mediated mechanisms. Inhibition experiments with specific blocking antibodies for the CLRs located on the surface of DCs revealed that mannose receptor and DC-SIGN (around 26% and 16%, respectively) are the main receptors contributing to the PM uptake by hmoDCs [18]. This enhanced uptake could help to increase the effective dose of the vaccine without increasing the allergen concentration, which might well help to design more effective and shorter immunotherapy protocols [6]. HmoDCs stimulated with N, P or PM express similar surface levels of HLA-DR or CD86. In contrast, PM-treated hmoDCs show higher levels of CD83 than N‑, but lower than P‑treated hmoDCs. Most importantly, PM treatment significantly increased the expression of the inhibitory molecule programmed death ligand 1 (PD-L1) both at protein and mRNA levels in hmoDCs [18]. PM-treated hmoDCs produced higher levels of IL-6 and IL-10 but lower IL-4 than N‑ or P‑treated DCs. These results were further confirmed in a human enriched total DC fraction containing both myeloid dendritic cells (mDCs) and plasmacytoid dendritic cells (pDCs). Interestingly, PM-treated mDCs induced higher IL-6 and IL-10 production than N‑ or P‑activated DCs, whereas pDCs stimulated with PM only produced IL-6 [18]. Supporting these data, PM-treated hmoDCs and PBMCs from healthy donors and allergic patients generated Th1 and Treg cells producing IFN-γ and IL-10, respectively, promoting a shift of Th2/Treg ratio in favor of Treg cells. The generated Treg cells were CD4^+^CD25^high^CD127^−^FOXP3^+^ and inhibited the proliferation of autologous PBMCs in a dose-dependent manner, showing a more potent suppression capacity than Treg cells generated by N‑ or P‑treated hmoDCs. The blocking of PD-L1 reduced the percentage of the induced FOXP3^+^ Treg cells, and the remaining Treg cells showed a lower suppression capacity [18]. Previous studies also demonstrated that PD-L1 ligation can lead to differentiation into FOXP3^+^ Treg cells [35, 36]. As previously discussed, mild oxidation of PM destroys the mannan structure, which significantly reduced PM uptake by hmoDCs. Similarly, oxidation significantly inhibits PD-L1 expression in hmoDCs, reduces IL-10 and IL-6 and increases IL-4 production and decreases the percentage of functional CD4^+^CD25^high^CD127^−^FOXP3^+^ Treg cells induced by PM-activated hmoDCs [18]. Collectively, all these data uncovered the importance of maintaining the integrity of the mannan structure to preserve the functional properties imprinted by allergoids conjugated to mannan to human DCs. Previous data targeting antigens to mannose receptor by coupling them to oxidized mannan reported protection against pathogenic autoimmunity by the expansion of antigen-specific anergic Th1 and Th17 cells without generating Treg cells [37]. In summary, preclinical assessment in human models demonstrated that allergoids conjugated to nonoxidized mannan might well represent novel suitable vaccines for AIT that are able to generate functional FOXP3^+^ Treg cells through mechanism partially depending on PD-L1 (Fig. 3b).

## Preclinical assessment of allergoids conjugated to mannan in animal models

Next, the *in vivo* immunogenicity of allergoids conjugated to nonoxidized mannan were assessed in different animal models. In rabbits, PM induced the production of potent IgG blocking antibodies (Fig. 3b; [18]). Humoral and cellular responses have been assessed in mice following subcutaneous or sublingual administration. By either route, PM was more efficient than N or P, to increase IgG2a/IgE and IFN-γ/IL-4 ratios, reflecting a Th1-type driven response. Importantly, an increase of FOXP3^+^ Treg and IL-10-producing cells was observed in mice immunized with PM as compared with P (Fig. 3b). While FOXP3^+^ Treg cells were readily detected in the spleen of mice upon subcutaneous immunization with PM [18], the sublingual route seemed less efficient in this regard and longer immunization protocols were required [23]. Interestingly, FOXP3^+^ Treg cells were also rapidly increased in the submandibular lymph nodes after sublingual immunization with PM [23]. PM were better captured by oral myeloid cells obtained from the sublingual tissue, including DCs (CD11b^+^) and macrophages (CD64^+^), than mannan-free allergens [23]. Remarkably, a better allergen uptake by these oral cells has been previously linked to an increase in regional Treg induction as well [38]. Supporting this view, upon mannan oxidation both the allergen uptake by oral cells and the induction of FOXP3^+^ Treg cells in regional lymph nodes were abolished [23]. Additional mechanisms triggered by PM on DCs, such as those described above in the human models, could also contribute to the generation of functional FOXP3^+^ Treg cells observed *in vivo*. The results with oral myeloid cells indicate that PM are better captured not only by hmoDCs but also by DC subsets obtained from mouse tissues in a steady-state situation [18, 23]. This feature has been also demonstrated with canine DCs derived from blood monocytes [26]. Similar to their human counterparts, canine DCs stimulated with PM also produced higher levels IL-10 and lower IL-4 than DCs activated with P or with PM subjected to further oxidation [26]. Of note, the studies with canine DCs were performed with allergoids conjugated to mannan derived from mites (*D. farinae*) allergens. Collectively, these data demonstrate that the better uptake of allergoids conjugated to nonoxidized mannan as well as the immunomodulating features exerted on DCs are observed for different allergen conjugates, DC subsets and animal species.

## Clinical assessment of allergoids conjugated to mannan

The first clinical experience with allergoids conjugated to mannan in AIT has been performed in veterinary medicine treating dogs with atopic dermatitis. Canine atopic dermatitis, the main manifestation of type I hypersensitivity in dogs, is a Th2-driven response to environmental allergens that may benefit of AIT [39]. The results of a pilot study using *D. farinae* allergoids conjugated to mannan administered subcutaneously are very encouraging as a clear clinical improvement was observed in most of the cases within the first 3 months of treatment (Gonzalez et al. submitted).

In humans, two dose-finding studies, for grass pollen and mites (NCT02654223 and NCT02661854, respectively, at www.clinicaltrials.gov), are currently ongoing in Spain (Fig. 3c). Each study consists of a double dummy with two arms to assess subcutaneous and sublingual administrations routes. The clinical outcome is rated by performing acoustic rhinometry measures in standardized nasal provocation tests. Similar to the clinical studies in dogs, the allergoids conjugated to mannan used for the subcutaneous administration is devoid of aluminum hydroxide. *In vitro* and *in vivo* data indicate that aluminum hydroxide impairs some of the key features induced by allergoids conjugated to nonoxidized mannan (manuscript in preparation). Therefore, subcutaneous and sublingual preparations are virtually identical, except for the glycerol excipient contained in the latter, thus, representing an interesting and paradigmatic study for comparison purposes.

## Conclusion and future perspectives

Allergoids conjugated to nonoxidized mannan might well represent next generation vaccines that enhance the allergen uptake by DCs and promote healthy immune responses to allergens. By targeting the allergoid to DCs through the conjugated mannan, the bioavailability of the administered dose would increase. Therefore, it is conceivable that less allergen is required per dose to obtain similar clinical efficacy than without mannan, or that efficacy increases at a similar dose because of this fact. This feature, together with the immunomodulatory properties reported for these glycoconjugates, makes allergoids coupled to mannan suitable novel vaccine candidates to improve current AIT protocols. Vaccines for grass pollen and mite allergy have been already developed, but the same concept is also being studied for other allergens such as *Olea*, *Betula*, *Ambrosia*, *Platanus* or peanut. Although all the available proof of concept and preclinical data point to the same direction, completion of phase II/III clinical trials is necessary to draw firm conclusions about the potential clinical performance of these novel vaccines targeting DCs.

## Abbreviations

AIT: Allergen-specific immunotherapy
CLR: C-type lectin receptor
DC: Dendritic cell
FOXP3: Forkhead box P3
hmoDCs: Human monocyte-derived dendritic cells
mDCs: Myeloid dendritic cells
MPL: Monophosphoryl lipid A
N: Native allergen extracts
ODN: Oligodeoxynucleotide
P: Polymerized allergoids
pDCs: Plasmacytoid dendritic cells
PD-L1: Programmed death-ligand 1
PM: Polymerized allergoids conjugated to mannan
TLR: Toll-like receptor
Treg: Regulatory T cells
VLP: Viral like particle
